# Pain detect questionnaire and pain catastrophizing scale affect gait pattern in patients with knee osteoarthritis

**DOI:** 10.1186/s40634-022-00492-w

**Published:** 2022-06-07

**Authors:** Kengo Harato, Yu Iwama, Kazuya Kaneda, Shu Kobayashi, Yasuo Niki, Takeo Nagura

**Affiliations:** Department of Orthopaedic Surgery, Keio University School of Medicine, 35 Shinanomachi, Shinjuku-ku, Tokyo, 160-8582 Japan

**Keywords:** Knee osteoarthritis, Gait analysis, Pain detect, Pain catastrophizing scale

## Abstract

**Purpose:**

Although pain phenotype affects clinical score in patients with knee osteoarthritis (OA), little information has been available on the relationship between pain phenotype and gait analysis. The purpose was to investigate the relationship between pain phenotype and gait parameters.

**Methods:**

A total of 34 patients (24 females and 10 males) with end-stage medial compartmental knee OA participated. All the patients were evaluated based on pain detect questionnaire (PD-Q) and pain catastrophizing scale (PCS). They were divided into two categories: Group Low (PD-Q score ≤ 12) and Group High (PD-Q score > 12), PCS + (PCS ≥ 23) and PCS- (PCS < 23). Gait analysis was performed using three-dimensional motion analysis system. Statistical analysis was done to compare gait parameters between groups for each allocation of PD-Q or PCS, separately.

**Results:**

Peak vertical ground reaction forces in Group Low and High were 0.99 ± 0.054 and 0.82 ± 0.17, respectively (*P* = 0.015). Peak knee adduction moments in Group Low and High were 0.70 ± 0.19 and 0.39 ± 0.14, respectively (*P* = 0.0022). For PCS allocation, knee extension limitation during mid-stance during gait were significantly larger in PCS- (*P* = 0.038).

**Conclusions:**

Patients with high PD-Q score had atypical gait pattern with smaller peak vertical ground reaction force and knee adduction moment, compared to patients with low PD-Q score. Moreover, patient with low PCS had different gait pattern in extension limitation, compared to those with high PCS. PD-Q and PCS would affect gait pattern in patients with knee OA. Level of evidence: III.

## Introduction

End-stage knee osteoarthritis (OA) is one of major factors which negatively affect daily activities in the elderly people. Although one of main symptoms in patients with knee OA is subjective pain, radiographic findings do not always match subjective pain level [1, 2]. Therefore, pain phenotype has been a common topic in knee OA [3]. Recently, patient dissatisfaction is a highly disputed issue in total knee arthroplasty (TKA) [4, 5], though pain relief should be obtained by the surgical procedure. Based on previous literatures, preoperative pain detect questionnaire (PD-Q) and pain catastrophizing scale (PCS) can be beneficial tools to evaluate whether patients have typical OA-related pain or neuropathic pain. Moreover, pain phenotype is associated with postoperative clinical outcome including Knee Society Score or others [6–8].

As knee function is difficult to assess [9], gait analysis has been performed as a functional assessment tool in patients with knee OA. As described in previous gait analysis studies, patients with medial knee OA have increased external knee adduction moment (KAM) during walking [10–14]. However, little attention has been paid to the relationship between pain phenotype and gait analysis.

The purpose of the present study was to investigate and clarify the relationship between pain phenotype and gait analysis. It was hypothesized that pain phenotype would affect gait characteristics in patients with end-stage knee OA.

## Materials and methods

### Subjects

A total of 34 knees in 34 patients (24 females and 10 males), who were diagnosed as end-stage medial compartmental knee OA of at least grade 3 severities according to the Kellgren–Lawrence scale, participated in the present study. All the patients were scheduled to undergo knee replacement surgery at our university hospital based on two surgeons’ judgement (KH and YN). Surgeries were done from April 2015 to April 2017. None of the subjects had any history of major injuries to the trunk, symptomatic lumbar canal stenosis and hip osteoarthritis. Inclusion criteria was as follows; walk without cane use and read the questionnaire without any assistance. Rheumatoid arthritis, osteonecrosis, and posttraumatic arthritis were excluded from the present study. A written informed consent form approved by Institutional Review Board of our university was obtained in each subject.

### Clinical score and patient-reported assessment

All the patients were evaluated based on 2011 New Knee Scoring System (KSS), PD-Q and PCS. PD-Q is a validated scale to assess neuropathic pain (NP). Scores range from 0–35, with PD-Q score ≤ 12; no NP (Low score), PD-Q score > 12; possible NP and PD-Q score ≥ 19; likely NP (Intermediate and High score) [15]. Patients were divided into two categories (Group L; Low, Group I & H; Intermediate and High) based on PD-Q. PCS was evaluated using total score and subcategories (rumination, helplessness, magnification). Patients were also divided into two categories (PCS- and PCS +) using total PCS. Cut-off value of total PCS was set at 23 points based on a previous study [16]. A PCS score ≥ 23 was classified as “high catastrophizing.”

### Gait analysis

**Fig. 1:**
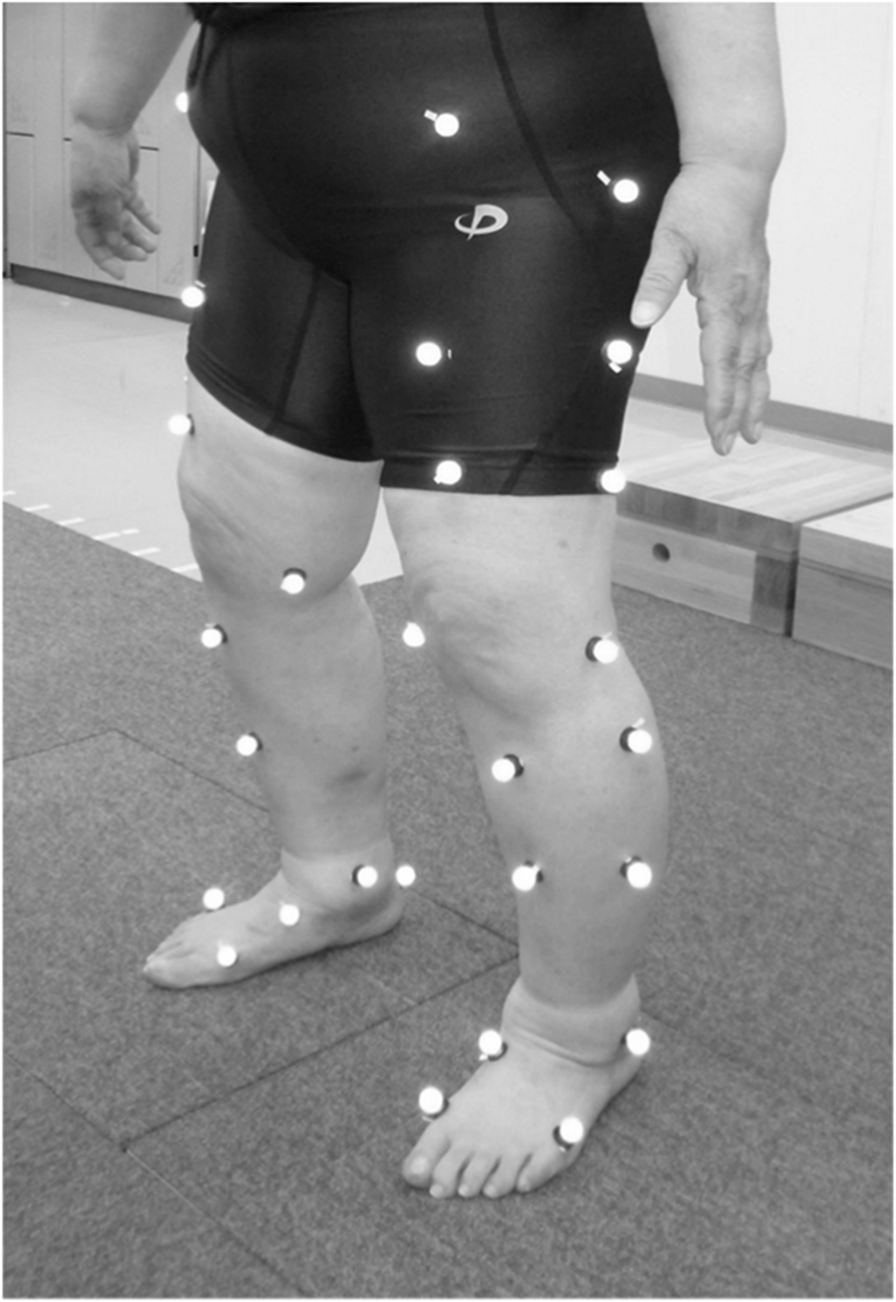
Gait analysis using a motion analysis system in patients with knee osteoarthritis

After a written informed consent was obtained, gait analysis was performed approximately one month before the surgery using three-dimensional motion analysis system which consisted of 8 cameras (120 frames/s; Oqus, Qualisys, Sweden) and 2 force plates (frequency 600 Hz; AM6110, Bertec, Columbus, OH, USA). A total of 46 retroreflective markers (14 mm in diameter) were placed on standardized bony landmarks (Fig. 1) [17, 18]. The force plate collected ground reaction force (GRF) data at 600 Hz were synchronized to the camera sampling rate (120 Hz). The subjects performed level walking at a self-selected speed. The motion of markers was recorded by Qualisys Track Manager Software (version 2.7). Visual 3D (C-motion Company, Rockville, MD, USA) was used to calculate knee kinematics and kinetics. Following gait parameters were assessed in the current investigation; gait speed (m/s), step length (m), single leg stance time (s), peak values of vertical GRF (kN/kg), flexion and extension limitation angles during midstance phase, and peak values of net external knee flexion and adduction moment (Nm/kg).

### Statistical analysis

After Shapiro–Wilk test was performed to confirm normality assumption, two-tailed unpaired t-test or Mann–Whitney U-test was used to compare age, BMI, knee scores, and gait parameters between Group L and I&H, and between PCS- and PCS + , respectively. Fisher exact test was used for categorical variables. P-values of < 0.05 were considered as significant. Thereafter, Bonferroni correction was done for selected mean values. In addition, concerning gait parameters, 95% confidence interval for the difference between groups was assessed both for PD-Q and PCS allocations. All statistical analyses were done with the Microsoft Excel Statistical Package, version 2015 (Social Survey Research Information, Tokyo). A power analysis was performed using G*Power (v3.1.9.2, Heinrich-Heine University, Düsseldorf, Germany). In terms of allocation based on PD-Q, effect size (d) was calculated using mean value and standard deviation of peak knee adduction moment in both groups. Thereafter, using a large effect size of 1.6 for Mann–Whitney U-test, a sample size of 4 and 10 for each group was estimated (β = 0.80, α = 0.05).

## Results

### Patient demographics, clinical score and patient-reported assessment

Mean age was 73.2 ± 7.2 years and mean body mass index (BMI) was 24.4 ± 2.5 kg/m^2^ for all patients. Twenty-nine patients were allocated to Group L, and five patients were to Group I & H. In addition, fourteen patients were allocated to PCS- and twenty patients were to PCS + . Age, BMI, and gender were not significantly different between groups both for PD-Q and PCS (Table 1). For PD-Q allocation, objective knee score, symptom score, satisfaction score, expectation score, and functional activity score in KSS and total PCS were not significantly different between groups (Table 2). For PCS allocation, objective knee score, satisfaction score, expectation score, and functional activity score in KSS were also not significantly different between groups, while symptom score as well as pain during gait were significantly worse in PCS- (Table 3).

**Table 1:** Patient demographics based on PD-Q and PCS (mean ± SD)

	PD-Q			PCS		
	Group L	Group I&H	*P* Value^a^	PCS-	PCS +	*P* Value^a^
Age (yrs)	74.2 ± 7.6	73.2 ± 7.1	n.s	72.1 ± 7.3	74.1 ± 7.0	n.s
BMI (kg/m^2^)	24.4 ± 2.0	24.4 ± 2.4	n.s	24.6 ± 2.3	24.3 ± 2.6	n.s
Gender (Female/male)	20 / 9	4 / 1	n.s	10 / 4	14 / 6	n.s

^a^Values obtained using two-tailed unpaired t-test or Mann–Whitney U test for age and BMI, and using Fisher exact test for gender distribution

**Table 2:** Clinical score and patient-reported assessment in each group based on PD-Q (mean ± SD)

	Group L	Group I & H	*P* Value^a^
Total PD-Q	4.7 ± 3.6	19.0 ± 2.2	< 0.001
Total KSS	123.5 ± 32.4	128.6 ± 33.5	n.s
Objective Knee Score	27.8 ± 9.1	35.4 ± 7.9	n.s
Range of motion	21.3 ± 4.9	24.4 ± 4.3	n.s
Flexion contracture	-6.9 ± 4.8	-4.0 ± 3.7	n.s
Symptom Score	10.0 ± 4.9	6.0 ± 4.1	n.s
Pain during gait	5.0 ± 2.7	2.4 ± 2.0	n.s
Satisfaction Score	16.5 ± 6.7	12.4 ± 6.7	n.s
Expectation Score	14.1 ± 1.2	13.2 ± 1.5	n.s
Functional Activity Score	55.1 ± 20.7	61.6 ± 21.3	n.s
Total PCS	24.0 ± 11.6	31.8 ± 11.9	n.s
Rumination (PCS)	12.8 ± 4.5	13.6 ± 5.6	n.s
Helplessness (PCS)	6.7 ± 5.1	12.0 ± 5.0	n.s
Magnification (PCS)	4.5 ± 3.2	6.2 ± 2.1	n.s

^a^Values obtained using two-tailed unpaired t-test or Mann–Whitney U test

**Table 3:** Clinical score and patient-reported assessment in each group based on PCS (mean ± SD)

	PCS- (< 23)	PCS + (≥ 23)	*P* Value^a^
Total PCS	13.6 ± 5.5	34.2 ± 6.9	< 0.0001
Total KSS	133.8 ± 37.2	119.0 ± 26.9	n.s
Objective Knee Score	28.5 ± 10.6	33.9 ± 6.4	n.s
Range of motion	20.9 ± 5.9	22.5 ± 3.6	n.s
Flexion contracture	-5.6 ± 5.2	-2.9 ± 3.3	n.s
Symptom Score	12.6 ± 4.5	6.9 ± 4.1	0.0020
Pain during gait	6.1 ± 2.4	3.4 ± 2.7	0.0099
Satisfaction Score	17.4 ± 7.2	14.2 ± 5.8	n.s
Expectation Score	14.1 ± 1.1	14.0 ± 1.3	n.s
Functional Activity Score	61.2 ± 22.4	50.1 ± 18.9	n.s
Total PD-Q	24.0 ± 11.6	31.8 ± 11.9	n.s

^a^Values obtained using two-tailed unpaired t-test or Mann–Whitney U test

### Gait parameters

For PD-Q allocation, peak vertical GRF was significantly greater in Group L than in Group I & H (*P* = 0.015, 95% confidence interval [0.00661–0.2509]). Besides, knee adduction moment was also significantly greater in Group L than in Group I & H (*P* = 0.0022, 95% confidence interval [0.0822–0.4445]), while other parameters including gait speed, step length, and stance time were not significantly different between groups (Table 4). For PCS allocation, knee extension limitation was significantly larger in PCS- than in PCS + (*P* = 0.038, 95% confidence interval [0.4436–14.5442]), whereas other parameters were not significantly different between groups (Table 5).

**Table 4:** Gait parameters in each group based on PD-Q (mean ± SD)

	Group L	Group I&H	*P* Value^a^
Gait speed (m/s)	0.79 ± 0.22	0.75 ± 0.20	n.s
Step length (m)	0.46 ± 0.15	0.45 ± 0.1	n.s
Stance time (s)	0.73 ± 0.21	0.72 ± 0.10	n.s
Peak vGRF (kN/kg)	0.99 ± 0.054	0.82 ± 0.17	0.015
Peak flexion during mid-stance (°)	13.6 ± 9.3	9.9 ± 9.8	n.s
Extension limitation during mid-stance (°)	8.4 ± 9.4	1.8 ± 8.4	n.s
Flex-ext excursion during mid-stance (°)	5.2 ± 3.9	8.1 ± 3.7	n.s
Peak knee flexion moment (Nm/kg)	0.21 ± 0.22	0.16 ± 0.20	n.s
Peak knee adduction moment (Nm/kg)	0.70 ± 0.19	0.39 ± 0.14	0.0022

^a^Values obtained using two-tailed unpaired t-test or Mann–Whitney U test

**Table 5:** Gait parameters in each group based on PCS (mean ± SD)

	PCS- (< 23)	PCS + (≥ 23)	*P* Value^a^
Gait speed (m/s)	0.70 ± 0.19	0.84 ± 0.22	n.s
Step length (m)	0.42 ± 0.17	0.48 ± 0.11	n.s
Stance time (s)	0.72 ± 0.27	0.71 ± 0.14	n.s
Peak vGRF (kN/kg)	0.99 ± 0.03	0.94 ± 0.12	n.s
Peak flexion during mid-stance (°)	15.8 ± 8.3	10.3 ± 9.2	n.s
Extension limitation during mid-stance (°)	11.3 ± 8.1	4.2 ± 8.9	0.038
Flex-ext excursion during mid-stance (°)	4.4 ± 3.1	6.2 ± 4.3	n.s
Peak knee flexion moment (Nm/kg)	0.22 ± 0.17	0.17 ± 0.24	n.s
Peak knee adduction moment (Nm/kg)	0.62 ± 0.18	0.63 ± 0.24	n.s

^a^Values obtained using two-tailed unpaired t-test or Mann–Whitney U test

## Discussion

The present results supported the hypothesis that pain phenotype would affect gait pattern in patients with symptomatic end-stage knee OA. The most important finding of the present study was that patients with high PD-Q score had atypical gait pattern with smaller peak vGRF and KAM, compared to patients with low PD-Q score.

Clinically, several knee scoring systems have been used to evaluate subjective pain in patients with knee OA. For instance, PD-Q has been a useful tool to evaluate a self-reported measure with the purpose of classifying patients as having either unlikely, uncertain or likely neuropathic pain components [19, 20]. In addition, PCS has been widely used to assess negative mental state that arises in the context of actual or anticipated pain, leading to a tendency to excessive worrying and the amplification of the sensation of pain [21]. Patients with high catastrophizing scale reported greater disability than those with low catastrophizing, with no differences as to pain intensity [16]. Recently, high rate of patient dissatisfaction is an important topic after total knee arthroplasty (TKA) [4, 5]. Based on previous literatures, preoperative PD-Q and PCS can be beneficial tools to evaluate whether patients have typical OA-related pain or NP. Forsythe ME et al. investigated a total of 55 patients with a primary diagnosis of knee OA, who were scheduled to undergo TKA, and concluded that preoperative PCS scores were shown to predict chronic postoperative pain at 24 months after TKA [22]. In addition, Warner SC et al. evaluated a total of 1151 patients with hip or knee OA before and after the surgery, and suggested that PD-Q appeared to be a useful tool in capturing factors that would contribute to postoperative satisfaction [23]. Some previous literatures have been available on the relationship between postoperative patient satisfaction and patient-reported questionnaire [24, 25]. However, correlation between gait analysis and pain phenotype based on PD-Q and PCS in patients with knee OA has not been investigated so far.

It has been well known that larger KAM is observed in early stance phase of patients with knee OA during gait [10–14]. Baliunas AJ et al. analyzed the gait of 31 knee OA patients and showed that peak values of KAM were significantly higher in knee OA group compared to healthy subjects [11]. In addition, patients with knee OA often show a typical stiffening gait pattern that improves dynamic knee stability to overcome joint laxity by decreasing knee flexion–extension excursion during mid-stance phase and increasing co-contraction of antagonist muscle [26, 27]. Similar gait pattern is known in patients with knee joint laxity due to ligament deficiency and has been reported as a risk of developing knee OA [28, 29]. In this study, despite the fact that subjects had at least grade 3 OA, patients with possible or likely NP (high PD-Q) did not show these typical gait characteristics of knee OA. Moreover, these patients also have atypical characteristics of OA-related pain, such as lower subjective pain during gait and higher helplessness score in PCS. Hochman et al. reported that depressive symptoms and pain catastrophizing may contribute to central sensitization, and were associated with the presence of NP symptoms [20]. Orthopaedic surgeons should take care of the prognosis of patients with such atypical OA-related pain and gait pattern.

PCS is a self-reported scale that assesses the level of catastrophizing in the presence of pain. It contains 13 items based on a Likert-type scale from 0 to 4. Higher scores represent higher levels of catastrophizing. It is divided into three domains: rumination, magnification and helplessness. According to a previous study, a score ≥ 23 was classified as “high catastrophizing,” whereas a score < 23 was classified as “low catastrophizing” [16]. In the present study, patient with low catastrophizing had larger symptom score in KSS including pain during gait and greater knee extension limitation during mid-stance of gait. Thus, patient with low PCS seemed to be a good candidate for surgery.

Several limitations should be described in the present study. First, all subjects in this study had end-stage OA and were candidates for TKA. Therefore, this result could be applied only for the patients with severe knee OA. Second, though the gait characteristics patients with OA were shown, the postoperative outcome of those patients is still unknown. Lastly, the sample size was different between Group L and Group I & H based on PD-Q. The power analysis presented was based on mean and standard deviation obtained from the dataset presented. Post-hoc power analyses might be inappropriate to detect true differences. However, to our knowledge, this is the first study to investigate correlation between gait analysis and patient-reported questionnaire (pain phenotype) in patients with knee OA. Therefore, the results of the current study are considered to contain useful information, when considering the correlation between gait characteristics and pain phenotype in patients with knee OA.

## Conclusions

Patients with possible and likely neuropathic pain (high PD-Q) had atypical gait pattern with smaller peak vGRF and KAM, compared to patients without NP (low PD-Q). Moreover, patient with low PCS had greater knee extension limitation during mid-stance in gait, compared to those with high PCS. Therefore, PD-Q and PCS would affect gait pattern in patients with end-stage knee OA. 

## Abbreviations

OA: Knee osteoarthritis
PD-Q: Pain detect questionnaire
PCS: Pain catastrophizing scale
KAM: Knee adduction moment
BMI: Body mass index
KSS: Knee Scoring System
NP: Neuropathic pain
GRF: Ground reaction force
TKA: Total knee arthroplasty
